# Functional interaction between S100A1 and MDM2 may modulate p53 signaling in normal and malignant endometrial cells

**DOI:** 10.1186/s12885-022-09249-1

**Published:** 2022-02-18

**Authors:** Mayu Nakagawa, Shyoma Higuchi, Miki Hashimura, Yasuko Oguri, Toshihide Matsumoto, Ako Yokoi, Yu Ishibashi, Takashi Ito, Makoto Saegusa

**Affiliations:** Department of Pathology, Kitasato University School of Medicine, 1-15-1 Kitasato, Minami-ku, Sagamihara, Kanagawa 252-0374 Japan

**Keywords:** S100A1, MDM2, p53, Menstrual cycle, Endometrial carcinoma

## Abstract

**Background:**

S100A1 expression is deregulated in a variety of human malignancies, but its role in normal and malignant endometrial cells is unclear.

**Methods:**

We used endometrial carcinoma (Em Ca) cell lines to evaluate the physical and functional interaction of S100A1 with p53 and its negative regulator, mouse double minute 2 (MDM2). We also evaluated the expression of S100A1, p53, and MDM2 in clinical samples consisting of 89 normal endometrial and 189 Em Ca tissues.

**Results:**

S100A1 interacted with MDM2 but not p53 in Em Ca cell lines. Treatment of cells stably overexpressing S100A1 with Nutlin-3A, an inhibitor of the p53/MDM2 interaction, increased expression of p53-target genes including *p21*^*waf1*^ and *BAX*. S100A1 overexpression enhanced cellular migration, but also sensitized cells to the antiproliferative and proapoptotic effects of Adriamycin, a genotoxic agent; these phenotypes were abrogated when S100A1 was knocked down using shRNA. In clinical samples from normal endometrium, S100A1 expression was significantly higher in endometrial glandular cells of the middle/late secretory and menstrual stages when compared to cells in the proliferative phases; high S100A1 was also positively correlated with expression of MDM2 and p21^waf1^ and apoptotic status, and inversely correlated with Ki-67 scores. However, such correlations were absent in Em Ca tissues.

**Conclusion:**

The interaction between S100A1 and MDM2 may modulate proliferation, susceptibility to apoptosis, and migration through alterations in p53 signaling in normal- but not malignant-endometrial cells.

**Supplementary Information:**

The online version contains supplementary material available at 10.1186/s12885-022-09249-1.

## Background

Endometrial carcinoma (Em Ca) is the most prevalent malignancy of the female genital tract in developing countries [1, 2]. In Japan, the age-adjusted prevalence of Em Ca for women in 2014 was 16.0 per 100,000. The overall rate has increased four-fold in the past 30 years, with a particularly rapid increase in women under 40 years old [3, 4]. Although most Em Ca patients are diagnosed at an early stage, 15—20% of these tumors are advanced or recurrent diseases and are associated with a 5-year survival rate of 17% [5]. Therefore, there is an urgent need to identify novel biomarkers or therapeutic targets for diagnosis or treatment.

The S100 protein family is comprises multigene calcium-binding proteins of the EF-hand type and has more than 25 members, each encoded by separate gene clusters on chromosome 1q21. This region is prone to chromosomal rearrangements and is frequently rearranged in cancers [6, 7]. Although S100 family members show high similarity at the sequence and structural level, they are not functionally interchangeable [8–10]. The proteins modulate a plethora of biological processes including proliferation, migration and invasion, inflammation, and differentiation. These effects are mediated via the interaction of S100 protein with a variety of protein targets including the tumor suppressor, p53, and its negative regulator, mouse double minute 2 (MDM2) [11].

S100 expression is deregulated in several human malignancies including carcinomas of the head, neck, lung, and breast [8–10]. However, the precise pattern of S100 expression and biological activity varies in a stage- and subtype-specific manner [8–10]. In gynecologic malignancies, analysis of S100 family members may facilitate diagnosis of ovarian carcinoma and help to refine prognosis [12]. However, a greater understanding of their functional roles in normal and malignant endometrium is still required.

Based on our previous work [13, 14], we hypothesized that S100 family members might regulate endometrial glandular cell biology and tumorigenesis through modulation of the p53/MDM2 axis. To test this, we first examined the expression of several S100 family members in Em Ca cell lines, and interrogated the Cancer Genome Atlas (TGCA) data to determine whether S100 expression had prognostic significance in Em Ca. We subsequently focused on an association between S100A1 and p53/MDM2, and determined its functional impact on proliferation, apoptosis, and migration in normal and malignant endometrial cells.

## Methods

### Plasmids and cell lines

A FLAG-tagged S100A1 expression plasmid generated by polymerase chain reaction (PCR) was cloned into a p3xFLAG-CMV14 vector (Sigma-Aldrich Chemicals, St. Louis, MO, USA). S100A1-specific short hairpin RNA (shRNA) oligonucleotides were designed as described previously [14]. Single-stranded S100A1 oligonucleotides were annealed and then cloned into *BamH*1-*EcoRI* sites of RNAi-Ready pSIREN-RetroQ vector (Takara, Shiga, Japan), according to the manufacturer’s instructions. The primer sequences for the PCR reaction used in this study are listed in Table 1.

**Table 1:** Primer sequences for functional analysis of *S100* gene family members used in this study

Assay	Gene		Sequence	size
RT-PCR (mRNA)	S100A1	Forward	5'-ATGGGCTCTGAGCTGGAGAC-3'	285 bp
		Reverse	5'-TCAACTGTTCTCCCAGAAGAAAT-3'	
	S100A2	Forward	5'-ATGATGTGCAGTTCTCTGGAGC-3'	297 bp
		Reverse	5'-TCAGGGTCGGTCTGGGCAGC-3'	
	S100A3	Forward	5'-AGTGAGGATGGCCAGGCCTCTG-3'	323 bp
		Reverse	5'-GGAGCAGAGGCTACTGGGAGCA-3'	
	S100A4	Forward	5'-GCCACCATGGCGTGCCCTCTGGAGAA-3'	306 bp
		Reverse	5'-TCATTTCTTCCTGGGCTGCTTATCTG-3'	
	S100A5	Forward	5'-ACACTGTGATGGAGACTCCTCT-3'	287bp
		Reverse	5'-TCACTTGTTGTCCTCTAGAAAGAAGT-3'	
	S100A6	Forward	5'-ATGGCATGCCCCCTGGATCAG-3'	273bp
		Reverse	5'-TCAGCCCTTGAGGGCTTCATT-3'	
	S100A7	Forward	5'-ATGAGCAACACTCAAGCTGAGAGGT-3'	306 bp
		Reverse	5'-TCACTGGCTGCCCCCGGAACAG-3'	
	S100A8	Forward	5'-ATGTTGACCGAGCTGGAGAAAG-3'	282 bp
		Reverse	5'-CTACTCTTTGTGGCTTTCTTCATGG-3'	
	S100A9	Forward	5'-ATGACTTGCAAAATGTCGCAGCTGG-3'	350 bp
		Reverse	5'-TGGTCTTAGGGGGTGCCCTCC-3'	
	S100A10	Forward	5'-CACCAAAATGCCATCTCAAATGG-3'	303 bp
		Reverse	5'-GCCTACTTCTTTCCCTTCTGCT-3'	
	S100A11	Forward	5'-ATGGCAAAAATCTCCAGCCCTACAG-3'	318 bp
		Reverse	5'-TCAGGTCCGCTTCGGGAAGGG-3'	
	S100A12	Forward	5'-ATGACAAAACTTGAAGAGCATCTGG-3'	279bp
		Reverse	5'-CTACTCTTTGTGGGTGTGGTAATGG-3'	
	S100A13	Forward	5'-ATGGCAGCAGAACCACTGAC-3'	297 bp
		Reverse	5'-TTACTTCTTCCTGATCTTCAGGTCT-3'	
	S100B	Forward	5'-ATGTCTGAGCTGGAGAAGGC-3'	279 bp
		Reverse	5'-TCACTCATGTTCAAAGAACTCGTGG-3'	
	CALB3(S100G)	Forward	5'-ATGAGTACTAAAAAGTCTCCTGAGGA-3'	256 bp
		Reverse	5'-TATTTTGTTTTCTCCTTCACTGGGA-3'	
	S100P	Forward	5'-ATGACGGAACTAGAGACAGCCAT-3'	288 bp
		Reverse	5'-TCATTTGAGTCCTGCCTTCTCAAAG-3'	
FLAG-tagged cDNA	S100A1	Forward	5'-CGCAAATGGGCGGTAGGCGTG-3'	
		Reverse	5'-GAACTGTTCTCCCAGAAGAA-3'	
shRNA	S100A1	Forward	5'-gatccgGAGACCCTCATCAACGTGTTttcaagagaAACACGTTGATGAGGGTCTCttttttg-3'	
		Reverse	5'-aattcaaaaaaGAGACCCTCATCAACGTGTTtctcttgaaAACACGTTGATGAGGGTCTCcg-3'	

Eight Em Ca cell lines (Hec1B, Hec6, Hec108, Hec116, Hec155, Hec251, Hec265 and Ishikawa) were used as described previously [15]. S100A1 expression plasmid or empty vector was transfected into Hec6 and Ishikawa cells (which lack endogenous S100A1 expression) and stable overexpressing clones were established. Conversely, we using an shRNA targeting the *S100A1* gene [13, 16] to reduce the levels of S100A1 in Hec251 cells, which have relatively high endogenous S100A1 expression (Additional file 3: Figure S3A). These cells are referred to as H251-S100A1-knockdown (KD) in the manuscript.

### Antibodies and reagents

Anti-FLAG M2 and anti-β-actin antibodies were purchased from Sigma-Aldrich Chemicals. Anti-p21^waf1^, anti-cyclin D1, anti-p53, anti-BCL2, and anti-Ki-67 antibodies were obtained from Dako (Glostrup, Denmark). Anti-p27^kip1^ and anti-BAX antibodies were from BD Biosciences (San Jose, CA, USA). Anti-MDM2 and anti-S100A1 were from Abcam (Cambridge, MA, USA). Anti-cleaved caspase-3 and anti-cleaved poly (ADP-ribose) polymerase 1 (PARP1)(Asp214)(D64E10) were from Cell Signaling Technology (Danvers, MA, USA). Anti-cyclin B1 and anti-cyclin A2 antibodies were purchased from Santa Cruz Biotechnology (Santa Cruz, CA, USA), and Novocastra (Newcastle, UK), respectively.

Rapamycin, aphidicolin, and nocodazole for synchronization of cells at G1, early S, and G2/M phases, respectively, were obtained from Calbiochem (Cambridge, MA, USA). Adriamycin (ADR) and Nutlin-3A were from Sigma-Aldrich Chemicals.

### Reverse transcription (RT)-PCR

cDNA was synthesized from 2 μg of total RNA. Amplification by RT-PCR was carried out in the exponential phase to allow comparison among cDNA synthesized from identical reactions using specific primers (Table 1). Primers for the *GAPDH* gene were also used as described previously [13, 16].

### Western blot assay and immunoprecipitation

Total cellular proteins were isolated using radioimmunoprecipitation assay (RIPA) buffer [20 mM Tris-HCl (pH 7.2), 1% Nonidet P-40, 0.5% sodium deoxycholate, 0.1% sodium dodecyl sulfate]. Cytoplasmic and nuclear fractions were prepared using ProteoExtract Subcellular Proteome Extraction kit (Merck KGaA, Darmstadt, Germany). Proteins were resolved by sodium dodecyle sulfate polyacrylamide gel electrophoresis (SDS-PAGE), transferred to membranes, and probed with primary antibodies coupled to the enhanced chemiluminescence (ECL) detection system (Amersham Pharmacia Biotechnology, Amersham, UK).

For immunoprecipitation, cells overexpressing FLAG-tagged S100A1 were lysed with immunoprecipitated (IP) buffer [10 mM Tris-HCl (pH 7.6), 100 mM NaCl, 10% NP-40] in the presence of 1 mM CaCl_2_. Cell lysates were cleared and incubated with anti-FLAG M2, anti-p53 or anti-MDM2 antibodies, followed by incubation with Protein G-Sepharose (Amersham Pharmacia Biotechnology). Western blot assay was subsequently performed with anti-FLAG M2, anti-p53, and anti-MDM2 antibodies.

### Flow cytometry

Cells were fixed using 70% alcohol and stained with propidium iodide (Sigma) for cell cycle analysis. Cells were analyzed by flow cytometry using BD FACS Calibur (BD Biosciences, Franklin Lakes, NJ, USA) and CellQuest Pro software version 3.3 (BD Biosciences).

### Apoptotic index

Apoptotic cells were identified in hematoxylin-eosin (HE)-stained sections according to the criteria of Kerr et al. [17]. Five fields were randomly selected, and the number of apoptotic cells was calculated by counting the mean number of apoptotic cells per high-power field (HPF).

### Immunofluorescence

After transfection of FLAG-tagged S100A1, the cells were incubated with anti-FLAG M2 antibody. FITC- or rhodamine-labeled anti-mouse or rabbit IgG secondaries (Molecular Probes, Leiden, The Netherlands) were used as described previously [13].

### Wound healing assay

Cells were seeded into 24-well tissue culture plates and grown to reach almost total confluence. After a cell monolayer formed, a scratch wound was made with a sterile 200-μL tip. The area of the wound was analyzed using ImageJ software version 1.41 (NIH, Bethesda, MD, USA). Cell migration was calculated based on the number of pixels occupied by the wound closure compared to control scratches.

### Migration assay

Cell migration was determined using 24-well transwell chambers with 8-μm pore size (Corning, NY, USA). The lower chamber was filled with medium containing 10% serum. Cells were suspended in serum-free medium and transferred into the upper chamber. After 48 h, the number of HE-stained cells on the bottom surface of the polycarbonate membranes was counted using a light microscope.

### Clinical cases

Histological findings were reviewed in hysterectomy specimens of endometrioid-type Em Cas from the case records of Kitasato University Hospital during the period from 2007 to 2020, according to the criteria of the 2014 World Health Organization classification [18]. Each case was also staged according to the 2009 International Federation of Gynecology and Obstetrics (FIGO) staging system and the TNM classification [19]. A total of 189 Em Ca cases, including 109 of grade (G)1, 51 of G2, and 29 of G3 were investigated. The mean age of the patients was 59.1 years (range from 31 to 92), and 113 were post-menopausal. In addition, our cases included 136 subcategorized as clinical FIGO stage I and 40 as stage II-IV, 77 with upper (<1/2) myometrial invasion and 98 with lower (≧1/2) myometrial invasion, as well as 20 that were positive for nodal metastasis and 156 that were negative.

Eighty-nine biopsy specimens of normal endometrial tissues including 32 in the proliferative phase, 41 in the secretory phase (18 early and 23 middle and late), and 16 in the menstrual phase were also investigated. All tissues were routinely fixed in 10% formalin and processed for embedding in paraffin. Approval for this study was given by the Ethics Committee of Kitasato University School of Medicine (B19-144).

### Immunohistochemistry (IHC)

IHC was performed using a combination of microwave oven heating and polymer immunocomplex (Envision, Dako) methods as described previously [15, 16].

For evaluation of IHC findings, scoring of cytoplasmic and/or nuclear immunoreactivity for S100A1, p53, MDM2, p21^waf1^, and Ki-67 in normal endometrial tissues was performed based on the percentage of immunopositive cells and the immunointensity; the values of these two parameters were multiplied together as described previously [13, 16]. In Em Ca tissues, scoring of S100A1 and p53 immunoreactivities was also carried out in a similar manner. Nuclear immunopositivity for MDM2, p21^waf1^, and Ki-67 was counted in at least 1000 cells from five randomly selected fields and the labeling indices (LIs) were then calculated as a percentage. In addition, the number of cleaved PARP1-positive cells in five randomly selected fields was used to calculate the mean number of apoptotic figures per HPF.

To examine any association between S100A1 immunoreactivity and several clinicopathological factors, the score was divided into two categories (high and low) with a mean score of 4.5 used as cutoff.

### Mutation analysis for *TP53* and *MDM2* genes

In Hec6, Hec251, and Ishikawa cells, exons 5 to 9 of the *TP53* gene were amplified by PCR, and the products were subjected to direct sequencing PCR as described previously [13]. Cancer Cell Line Encyclopedia (CCLE) data describing *MDM2* gene status was also extracted form cBioPortal (http://www.cbioportal.org).

### TCGA data analysis

Uterine Corpus Endometrial Carcinoma TCGA PanCancer data describing mRNA expression data (RNA Seq V2 PSEM) for the S100 family in 520 cases were extracted from cBioportal for Cancer Genomics (http://www.cbioportal.org/). The data were subcategorized into ‘high’ and ‘low’ groups (scores >0 and 0, respectively) based on the median Z score (= 0) for mRNA expression levels in each category, and then examined for any correlation with overall survival (OS) or progression-free survival (PFS).

### Statistics

Comparative data were analyzed using the Mann-Whitney *U*-test, and Spearman’s correlation coefficient. The cutoff for statistical significance was set as *P* < 0.05.

## Results

### Expression of S100 family members and their prognostic significance in Em Ca

We first examined the expression of 13 S100 family members in eight Em Ca cell lines. S100A10 and S100A11 mRNAs were the most abundant, whereas levels of S100A1, S100A2, S100A4, S100A9, and S100A13 were moderate. There was very little or no expression of S100A3, S100A5, S100A6, S100B, S100CA, and S100P (Additional file 1: Figure S1).

We then analyzed data from TCGA to determine whether any S100 mRNAs had prognostic significance in Em Ca. Among the 18 S100 family members, copy number variations including gain and amplification were significantly higher in high mRNA expression categories compared to the low groups in 11 S100 family members, whereas such associations with the gene mutations were not evident (Additional file 9: Table S1). The mRNA expression in 10 S100 family members were also significantly associated with *TP53*, but not *MDM2*, gene status (Additional file 2: Figure S2). In addition, Kaplan-Meier curve analysis showed that patients with high S100A1, S100A5, and S100P mRNA expression had poorer OS and/or PFS when compared to patients with low expression of these genes (Additional file 3: Figure S3). Based on the above findings, we therefore focused on the functional role of S100A1 in normal and malignant endometrial cells.

### S100A1 interacts with the p53/MDM2 axis in Em Ca cells

Three Em Ca cell lines, Hec6, Hec251, and Ishikawa cells, had mutations in the DNA binding domains of the *TP53* gene (Additional file 4: Figure S4). CCLE data analysis also revealed *MDM2* gene mutation (P301T in the acidic domain) in the Hec251 cell line, whereas the mutations were not evident in the Hec6 and Ishikawa cell lines.

Some S100 family members are known to modulate the p53/MDM2 axis [11]. To examine the association between S100A1, p53, and MDM2 in Em Ca cells, we established cell lines stably overexpressing FLAG-tagged S100A1 in Hec6 cells (clones H6-S100A1#55 and #58) and Ishikawa cells (clones Ish-S100A1#14 and #23). Conversely, we also generated two independent Hec251 clones in which S100A1 expression was blocked by an S100A1-specific shRNA (H251-shS100A1#3 and #8) (Additional file 5: Figure S5).

**Fig. 1:**
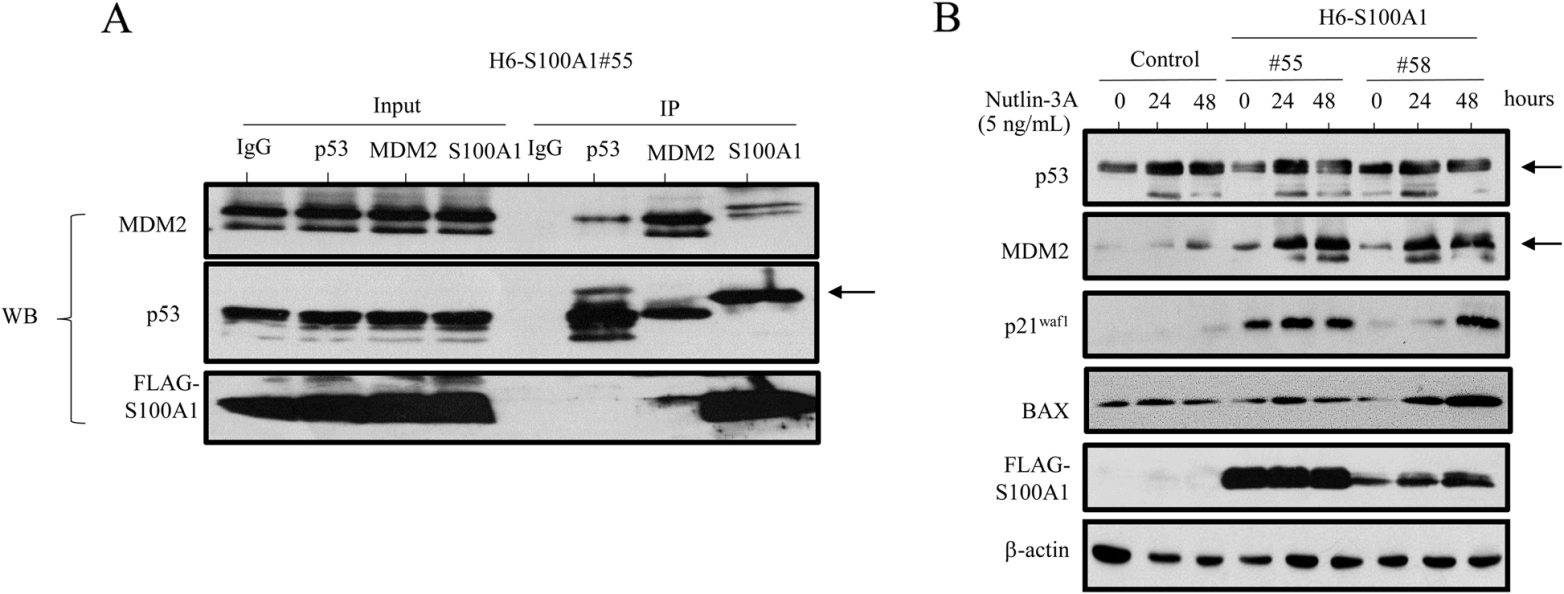
Interactions between S100A1, MDM2, and p53 in Hec6 cells. **A** Western blot analysis (WB) with anti-MDM2 (upper panel), anti-p53 (middle panel), and anti-FLAG M2 antibodies (lower panel) after immunoprecipitation (IP) with the indicated antibodies using stable H6-S100A1#55 cell lysates. Input represents 5% of the total cell extract. Normal rabbit IgG was used as a negative control. In the middle panel (p53), the band indicated by an arrow in the S100A1 lane is non-specific, since the molecular weight is slightly higher than that of endogenous p53 (input). **B** Western blot analysis of the indicated proteins in H6-S100A1 cells in response to Nutlin-3A treatment for the times shown. The p53 and MDM2 bands are indicated by arrows

We were able to detect reciprocal pairwise interactions between S100A1, p53, and MDM2 in both stable H6- and Ish-S100A1 cell lines, with the exception of an association between S100A1 and p53 (Fig. 1A and Additional file 6: Figure S6A). Treatment of cells with Nutlin-3A, which inhibits the proteasomal degradation of p53 by antagonizing its interaction with MDM2 [20], also increased p53 and MDM2 protein levels. This was accompanied by an increase in the expression of p53 transcriptional targets including p21^waf1^ and/or BAX in S100A1 overexpressing cells as compared to control cells (Fig. 1B and Additional file 6: Figure S6B). These findings suggest that S100A1 interacts with MDM2, leading to induction of p53-target molecules in Em Ca cells.

### Overexpression of S100A1 is associated with changes in cell proliferation, apoptosis, and migration in Em Ca cells

**Fig. 2:**
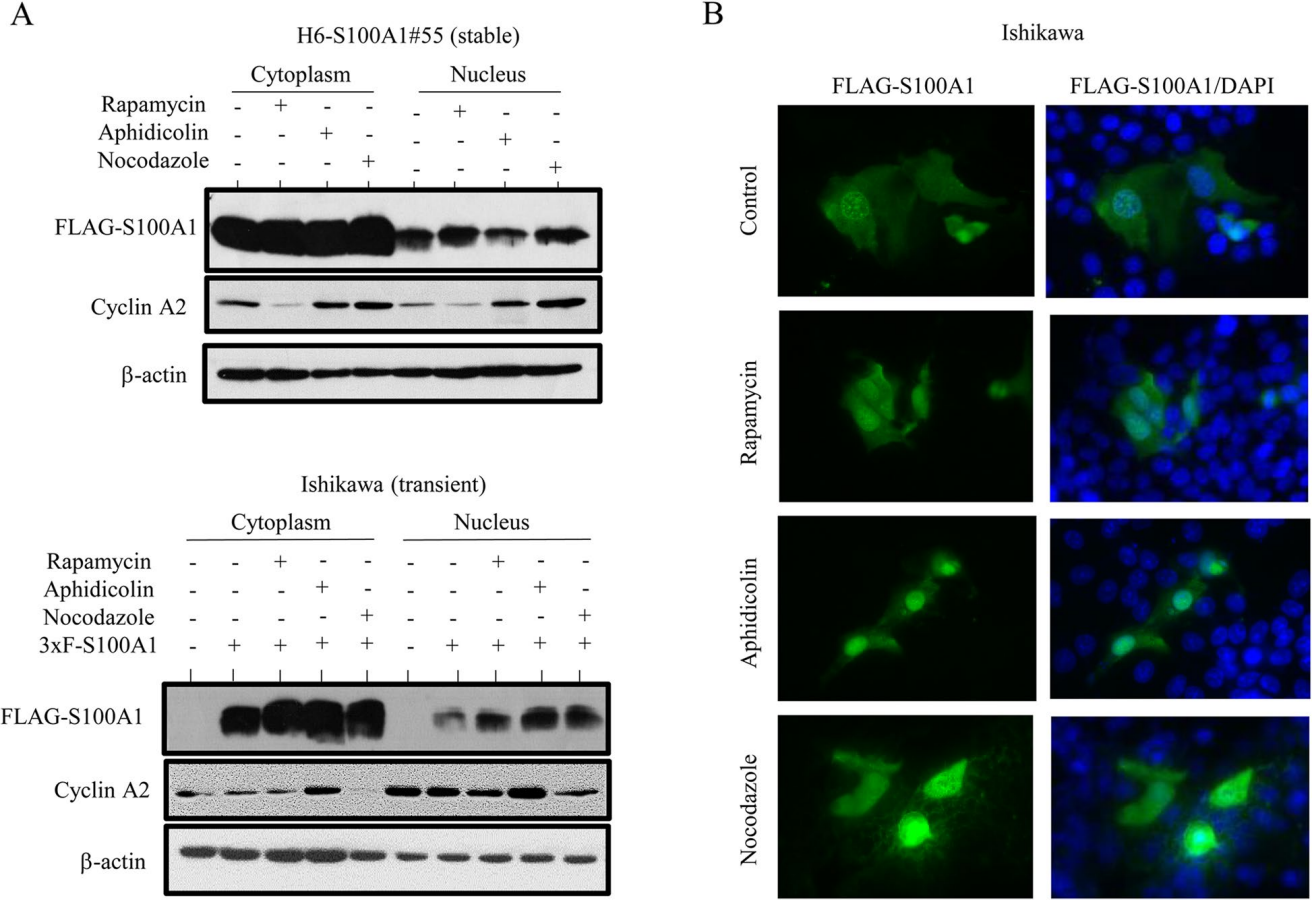
S100A1 expression during cell cycle progression in Hec6 and Ishikawa cells. **A** Stable H6-S100A1#55 (upper) and S100A1-transiently transfected Ishikawa cells for 24 h (lower) were synchronized in the G1 phase by treatment with 50 nM rapamycin, in the early S phase by 2 μg/mL aphidicolin, or in the G2/M phase by 0.25 μg/mL nocodazole for 24 h. Western blot analysis of proteins in the cytoplasmic and the nuclear fractions. **B** After FLAG-tagged S100A1 was transiently transfected into Ishikawa cells, the cells were treated with 50 nM rapamycin, 2 μg/mL aphidicolin, or 0.25 μg/mL nocodazole for 24 h and then stained using anti-FLAG M2 antibody. Note the cytoplasmic and nuclear S100A1 staining, independent of the cell cycle phase

**Fig. 3:**
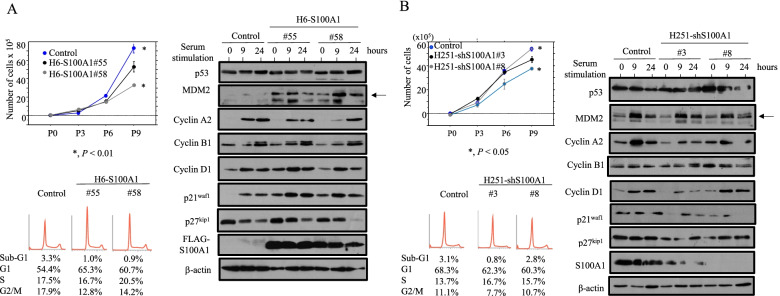
Changes in proliferation following overexpression and knockdown of S100A1 in Hec6 or Hec251 cells. **A**, **B** Upper left: two independent clones of stable H6-S100A1 (A) and H251-shS100A1 cells (**B**), as well as controls, were seeded at low density. The cell numbers are presented as mean ± SDs. P0, P3, P6, and P9 are 0, 3, 6, and 9 days after cell passage, respectively. Lower left: FACS analyses of stable H6-S100A1, H251-shS100A1 and control cells at 3 days after seeding (P3). Right: western blot analysis of the indicated proteins in stable H6-S100A1 cells (**A**), H251-shS100A1 cells (**B**), and controls following re-stimulation of serum-starved (24 h) cells with 10% serum for the indicated times. The p53 and MDM2 bands are indicated by arrows

To examine whether S100A1 modulates proliferation in Em Ca cells, we looked for variations in S100A1 expression during cell cycle progression. Figure 2A shows that the nucleocytoplasmic distribution of S100A1 in cells stably or transiently overexpressing the protein was similar in G1-, early S-, and G2/M-arrested cells; this was consistent with data from immunofluorescence staining (Fig. 2B). To further examine whether S100A1 could alter cell growth and expression of major cell cycle-related molecules in G1, S, and G2/M phases during cell cycle progression, we seeded S100A1-overexpressing and S100A1-KD cells at low density and rendered them quiescent by serum starvation before adding back serum to induce cell cycle entry. As shown in Fig. 3A (and Additional file 7: Figure S7A), cells stably overexpressing S100A1 had a low proliferative rate, particularly in the exponential growth phase, along with an increased proportion of cells in G1 phase during cell cycle progression; this was concomitant with increased MDM2 and p21^waf1^ expression. In contrast, H251-S100A1-KD cells proliferated more rapidly and this resulted in a decrease in the numbers of cells in G1 phase, together with decreased p21^waf1^ but not MDM2 expression (Fig. 3B).

**Fig. 4:**
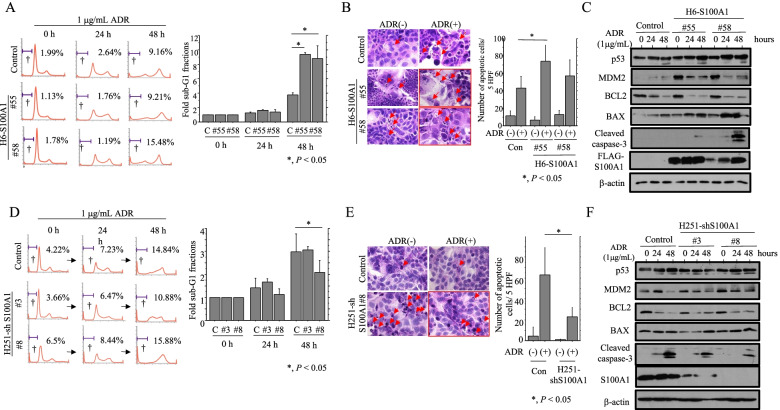
Changes in apoptosis following overexpression or knockdown of S100A1 in Hec6 or Hec251 cells. **A**, **D** Left: stable H6-S100A1 (A), H251-shS100A1 cells (**D**), and controls were treated with 1 μg/mL Adriamycin (ADR) for the time shown. Daggers indicate sub-G1 fraction. Right: the percentages of cells undergoing apoptosis (sub-G1 fractions) were calculated following flow cytometry. C, control (**B**, **E**) Left: after 1 μg/mL ADR treatment, stable H6-S100A1 (**B**), H251-shS100A1 cells (**E**), and controls undergoing apoptosis are indicated by arrows. Original magnification, x400. Right: the numbers of apoptotic cells are shown as mean ± SDs. Con, control (**C**, **F**) Western blot analysis of the indicated proteins in total lysates from stable H6-S100A1 cells (**C**), H251-shS100A1 cells (**F**), and controls treated with 1 μg/mL ADR for the times shown

Next, we examined whether there was an association between S100A1 expression and apoptosis in response to the genotoxin, ADR. Overexpression of S100A1 in Hec6 cells increased the sub-G1 fraction (Fig. 4A) and the number of apoptotic cells (Fig. 4B) following ADR treatment. This was observed together with increased expression of p53, MDM2, BAX, and cleaved caspase-3, and with decreased BCL2 expression (Fig. 4C). Similar findings were also observed in ADR-treated Ish-S100A1 cells (Additional file 7: Figure S7B, C, D). In contrast, the sub-G1 fraction and the number of apoptotic cells was reduced when S100A1 levels were reduced with shRNA (Fig. 4D, E). Consistent with this, levels of cleaved caspase-3 were lower in ADR-treated H251-S100A1-KD cells (Fig. 4F).

**Fig. 5:**
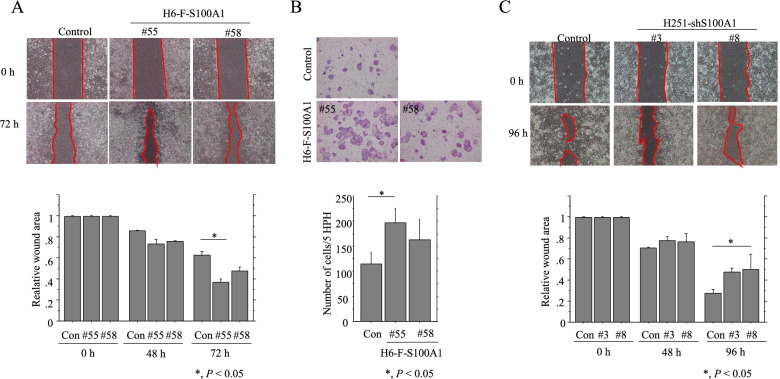
Changes in migration following overexpression or knockdown of S100A1 in Hec6 and Hec251 cells. **A**, **C** Upper: a scratch was made in the middle of a layer of confluent H6-S100A1 (**A**) or H251-shS100A1 cells (**C**), and phase contrast images were taken 72 h or 96 h later. The red lines indicate borderlines between confluent cell layers and wound area. Lower: the wound areas were calculated using Image J software version 1.41, with the area at 0 h post-wounding set as 1. The experiments were performed in triplicate. Data are expressed as mean ± SDs. **B** Upper panel: stable H6-S100A1 cells and control cells were seeded in a 24-well transwell plate and incubated for 48 h in serum-free medium. Cells that migrated were stained with hematoxylin-eosin and counted using a light microscope. Lower panel: cell numbers are presented. The experiment was performed in duplicate. Con, control

Since several S100 proteins modulate cell mobility [21–23], we examined whether S100A1 specifically regulated cell migration. Overexpression of S100A1 significantly increased the migration rate of cells in a wound assay in both cell types studied (Fig. 5A, B and Additional file 8: Figure S8). In contrast, cellular migration capability was significantly decreased when S100A1 was knocked down (Fig. 5C).

These findings suggest that S100A1 modulates proliferation, apoptosis, and migration in Em Ca cells.

### IHC findings in normal and malignant endometrial tissue

**Fig. 6:**
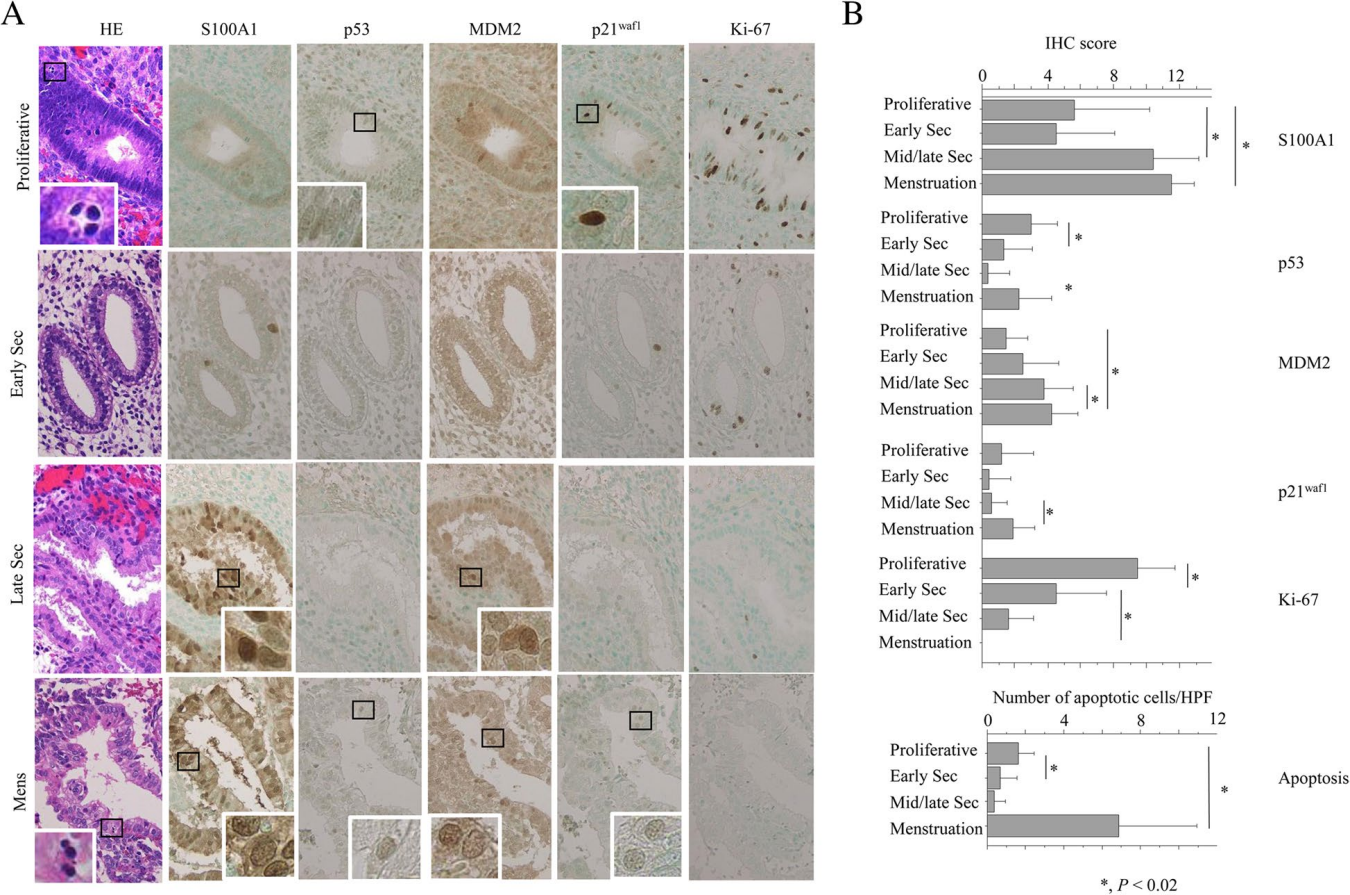
Immunohistochemistry (IHC) findings in serial sections of normal endometrium. **A** Staining with hematoxylin-eosin (HE) and IHC for the indicated proteins in proliferative, early and late secretory (Sec), and menstrual (Mens) stages. Closed boxes are magnified in the insets. In the HE panels, apoptotic cells are shown in the insets. Original magnification, x200 and x400 (inset). **B** IHC score for the indicated molecules and number of apoptotic cells in normal endometrial glandular cells during the menstrual cycle. Data are expressed as mean ± SDs. Mid/late Sec, middle and late secretory endometrium.

In the glandular components of normal endometrium, we observed nuclear/cytoplasmic S100A1 immunostaining and nuclear immunoreactivity for p53, MDM2, p21^waf1^, and Ki-67 (Fig. 6A). Average IHC scores for S100A1, MDM2, and p21^waf1^ and apoptotic features were significantly higher in menstrual and/or middle/late secretory phases than in other stages. In contrast, Ki-67 score showed a significant stepwise decrease from proliferative to menstrual phases (Fig. 6B). As shown in Table 2, average S100A1 score positively correlated with MDM2 and p21^waf1^ score and with apoptotic features; S100A1 score was inversely correlated with Ki-67 score. p53 score was significantly associated with Ki-67 score and apoptotic status, whereas p21^waf1^ score was significantly associated with MDM2 score and apoptotic cells.

**Table 2:** Correlations among S100A1 and its related markers in normal and malignant endometrial lesions

	S100A1	p53	MDM2	p21^waf1^	Ki-67
	*ρ* (*P*)	*ρ* (*P*)	*ρ* (*P*)	*ρ* (*P*)	*ρ* (*P*)
Normal endometrium					
p53	0.12	*	*	*	*
	(0.4)				
MDM2	0.44	0.14	*	*	*
	(0.007)	(0.4)			
p21^waf1^	0.4	0.36	0.46	*	*
	(0.03)	(0.03)	(0.0006)		
Ki-67	-0.45	0.51	-0.06	0.08	*
	(0.04)	(0.03)	(0.7)	(0.7)	
Apoptosis	0.39	0.53	0.17	0.45	-0.11
	(0.06)	(0.001)	(0.28)	(0.006)	(0.6)
Malignant endometrium					
p53	0.2	*	*	*	*
	(0.06)				
MDM2	0.05	0.09	*	*	*
	(0.6)	(0.4)			
p21^waf1^	0.24	0.26	0.35	*	*
	(0.02)	(0.01)	(0.001)		
Ki-67	0.09	0.42	-0.06	0.36	*
	(0.3)	(<0.0001)	(0.6)	(0.0006)	
cPARP1	0.17	0.49	-0.14	0.27	0.66
	(0.1)	(<0.0001)	(0.2)	(0.01)	(<0.0001)

**Fig. 7:**
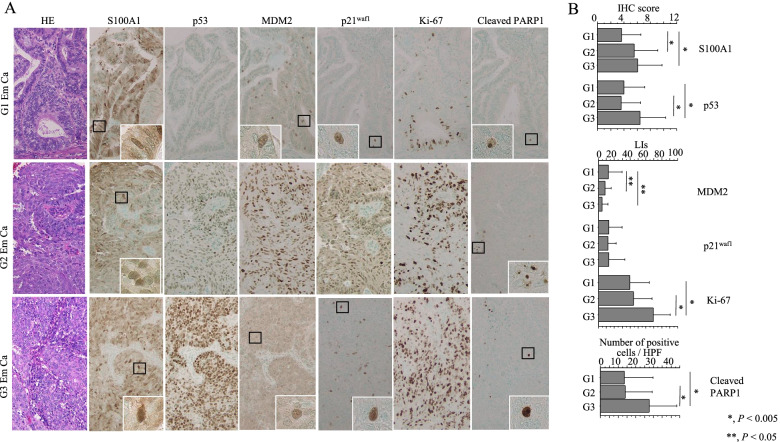
Immunohistochemistry (IHC) findings in serial sections of Em Ca tissues. **A** Staining with hematoxylin-eosin (HE) and IHC for the indicated proteins in G1 (upper), G2 (middle), and G3 (lower) Em Cas. Closed boxes are magnified in the insets. Original magnification, x200 and x400 (inset). **B** IHC score and labeling indices (LIs) for the indicated molecules and number of cleaved PARP1-positive cells in Em Ca tissues. Data are expressed as mean ± SDs

We also examined the coincidence of the above markers with cleaved PARP1, a marker of cells that are undergoing apoptosis [24], in Em Ca tissue. A variety of p53 immunointensities was observed in the tumor tissues, but such findings were relatively minor in those of other markers (Fig. 7A). S100A1 and p53 scores, Ki-67 LIs, and cleaved PARP1-positive cells were significantly higher in G3 Em Ca than G1 or G2 tumors, whereas MDM2 score was significantly higher in the latter (Fig. 7B). Average S100A1 score was not associated with any of the markers investigated, whereas p53 score positively correlated with Ki-67 LIs and cleaved PARP1-positive cells (Table 2). There were no associations between S100A1 score and any of the clinicopathological factors examined (see Methods and Additional file 10: Table S2).

## Discussion

We previously demonstrated that both cytoplasmic and nuclear S100A4 expression is substantially increased in G2/M-arrested Em Ca cells compared with G1- and early S-arrested cells [13]. Since the interaction between S100A1 and S100A4 is mutually antagonistic [25, 26], we expected cyclic S100A1 expression during cell cycle progression. However, our current data reveal that this is not the case. In general, S100A1 is abundantly expressed in cardiomyocytes, skeletal muscle fibers, and neural populations, where it regulates cytoskeletal fibers including microtubules, type III intermediate filaments, and actin [27]. Together with our current results, this therefore suggests that expression of S100A1 (and in particular the cytoplasmic form) may help to establish and maintain endometrial gland cell shape, which in turn promotes a secretory phenotype during normal menstrual cycles.

Although Hec6, Hec251, and Ishikawa cells used in this study had *TP53* mutations, a mutant p53 knock-in mouse in which normal tissues and some tumors have low levels of mutant p53 protein unless MDM2 or p16^INK4A^ are absent, indicating that the mutant p53 is regulated by the same signals as wild-type p53 [28]. Moreover, stabilization of mutant p53 nevertheless triggers extensive apoptosis, indicative of residual wild-type activities or p53-independent pathway activity [29, 30]. Thus, there is complexity of mutant p53, where a mutant can be anti-apoptotic in some carcinoma cells while pro-apoptotic in others.

Consistent with a previous study showing that S100A1, as well as S100A2,

S100A4, and S100A6, interact with MDM2 and disrupt the extent of MDM2-mediated p53 ubiquitylation [31], we found that S100A1 interacts with MDM2, the negative regulator of p53, in Em Ca cells. Moreover, treatment with the MDM2 antagonist, Nutlin-3A [20], selectively increased MDM2 expression in S100A1-overexpressing Em Ca cells. Since this treatment also elicited increased expression of p53 and its target genes including *p21*^*waf1*^ and *BAX*, we suggest that S100A1 overexpression may increase the cellular fraction of p53-free MDM2. Although some S100 proteins including S100B, S100A4, S100A2, and S100A1 also interact with p53, they exert different effects on p53 activity [9, 32, 33], indicating that the activity balance of different S100 proteins may contribute to determining the functions of S100 family members in cells. However, we could not demonstrate any interaction between S100A1 and p53 in Em Ca cells, whereas other studies have demonstrated that S100A1 binds to a peptide derived from the C-terminal negative regulatory domain of p53 [32, 33]. This discrepancy suggests that S100A1 may require additional cell- and/or tissue-specific factors to interact with p53. As another possibility, it appears that p53-S100A1 complex may be short lived or dependent on a high local concentration of the interactors or the intactness of the subcellular compartment as described previously [34]. In addition, there is a difference in detection sensitivity among the three antibodies used in this study. Similar findings were also observed regarding the association between p53 and S100A4 [14, 35].

In our results, stable overexpression of S100A1 significantly inhibited cell proliferation, and increased MDM2 and p21^waf1^ expression in response to serum stimulation. S100A1 overexpression also sensitized cells to genotoxin-induced apoptosis, probably due to increased BAX and cleaved caspase-3 expression and decreased BCL2 expression. In contrast, S100A1-KD cells elicited opposite effects. From these data, we infer that S100A1 may bind to MDM2 and reduce its ubiquitylation of p53, which in turn leads to reduced proliferation and increased apoptosis, probably due to transcriptional activation of p53-target genes including *p21*^*waf1*^ and *BAX*. In addition, S100A1 may interfere with MDM2-mediated p21^waf1^ proteasomal degradation through its interaction with MDM2 [36, 37]. Consistent with these, we found that the S100A1 score was significantly associated with MDM2 and p21^waf1^ expression, as well as apoptotic status, in normal endometrial tissue during the menstrual cycle. Interestingly, the relationship between MDM2, Murine Double Minute X, and their putative targets E2F1, E2F3a, and p70 also suggests a mechanism for regulation of the cell cycle in the absence of wild-type p53 [38].

We also found that S100A1 overexpression enhanced cell migration, whereas S100A1 knockdown reduced motility. These pro-migratory but antiproliferative activities of S100A1 are consistent findings that migratory cells have a lower proliferation rate than those in the less mobile but highly proliferative tumor core [39–41]. Similar findings were also observed in ovarian carcinoma cells, supporting observations that S100A1 plays an important role in tumor cell migration through interaction with different components of the cytoskeleton including microtubule and intermediate filaments [42]. MDM2 induces epithelial-mesenchymal transition by enhancing Snail expression or via activation of Smad2/3 signaling in breast and lung carcinomas [43, 44]. As these phenotypes are also induced by S100A1, and we observe a physical interaction between S100A1 and MDM2, we infer that S100A1 may mediate some of its biological effects via MDM2. Alternatively, it appears that overexpression or knockdown of S100A1 may be due to disruption of the S100A1-mediated physiological homeostasis in cells, and this in turn results in abnormalities of the p53/MDM2 axis.

S100A1 expression can have different prognostic significance dependent on the specific type of gynecological malignancy. For example, S100A1 serves as a favorable prognostic factor in endometrioid subtypes of ovarian carcinomas [45], whereas its expression is associated with poor prognosis in ovarian and endometrial endometrioid carcinomas [46]. We found that S100A1 expression was significantly higher in G3 Em Ca as compared to G1 tumors. Moreover, our TCGA data analysis revealed a significant association between S100A1 mRNA expression and poorer OS and PFS in Em Ca. We therefore speculate that high S100A1 expression is an indicator of poor prognosis in Em Ca. However, S100A1 expression level did not correlate with several clinicopathological factors or with the status of MDM2 and other molecules in the p53 pathway in Em Ca tissues. The discrepancies between the results of Em Ca cell line- and tissue-based analyses are probably due to the presence of microenvironmental factors in the latter. For example, interleukin (IL)-6 and IL-8 released from myofibroblasts in the tumor microenvironment induce myeloid cells to differentiate into S100A8/S100A9-expressing myeloid-derived suppressor cells and M2 macrophages [47]. Further studies are now required to more fully elucidate the influence of S100A1 in malignancies, particularly with regard to metastasis and immune surveillance of tumors.

## Conclusions

S100A1 expression may modulate cell proliferation, apoptosis and migration through its interaction with MDM2 during the menstrual cycle in normal, but not malignant, endometrial tissue.

## Supplementary Information

**Additional file 1: Figure S1.** S100 family member mRNA expression in Em Ca cell lines. Left: expression of 13 S100 family members in eight Em Ca cell lines detected by RT-PCR assay. Right: relative amounts of S100 mRNA calculated by normalization to signals for the *GAPDH* gene using ImageJ software version 1.41.

**Additional file 2: Figure S2**. Relationship between expression of S100 family members and TP53 and MDM2 gene status derived from TCGA Em Ca data analysis. Comparison of the mRNA status of S100 family members between wild- and mutant types of *TP53* (A) and *MDM2* genes (B). n, number of cases; Wt, wild-type; Mu, mutant type.

**Additional file 3: Figure S3.** Relationship between expression of S100 family members and prognosis derived from TCGA Em Ca data analysis. Overall survival (OS) and progression free survival (PFS) between low and high mRNA expression categories of 18 S100 family members.

**Additional file 4: Figure S4.** *TP53* gene status in Hec6, Ishikawa, and Hec251 cell lines. *, stop codon.

**Additional file 5: Figure S5.** Western blot analysis of the indicated proteins in total lysates from eight Em Ca cell lines (A), stable H6- and Ish-S100A1 cells (B), and H251-shS100A1 cells (C), as well as control cells (Con).

**Additional file 6: Figure S6**. Interactions between S100A1, MDM2, and p53 in stable Ish-S100A1 cells. (A) Western blot analysis (WB) with anti-MDM2 (upper panel), anti-p53 (middle panel), and anti-FLAG M2 antibodies (lower panel) after immunoprecipitation (IP) with the indicated antibodies using stable Ish-S100A1 cell lysates. Input represents 5% of the total cell extract. Normal rabbit IgG was used as a negative control. In the middle panel (p53), the band indicated by an arrow in the S100A1 lane is non-specific, since the molecular weight is slightly higher compared to that of endogenous p53. (B) Western blot analysis of the indicated proteins in stable Ish-S100A1 cells in response to Nutlin-3A treatment for the times shown. The p53 band is indicated by arrows.

**Additional file 7: Figure S7.** Changes in proliferation and apoptosis following overexpression of S100A1 in Ishikawa cells. (A) Upper left: two independent clones of stable Ish-S100A1 cells and control cells were seeded at low density. The cell numbers are presented as mean ± SDs. P0, P3, P6, and P9 are 0, 3, 6, and 9 days after cell passage, respectively. Upper right: FACS analyses of stable Ish-S100A1 and control cells at 3 days after seeding (P3). Lower: western blot analysis of the indicated proteins in stable Ish-S100A1 cells and controls following re-stimulation of serum-starved (24 h) cells with 10% serum for the indicated times. The p53 band is indicated by arrows. (B) Upper: stable Ish-S100A1 and control cells were treated with 1 μg/mL Adriamycin (ADR) for the times shown. Daggers indicate the sub-G1 fraction. Lower: the percentages of cells undergoing apoptosis (sub-G1 fractions) were calculated following flow cytometry. C, control (C) Left: after 1 μg/mL ADR treatment, stable Ish-S100A1 and control cells undergoing apoptosis are indicated by arrows. Original magnification, x400. Right: the numbers of apoptotic cells are shown as mean ± SDs. Con, control (D) Western blot analysis of the indicated proteins in total lysates from stable Ish-S100A1 and control cells treated with 1 μg/mL ADR for the times shown. The p53 band is indicated by arrows.

**Additional file 8: Figure S8**. Changes in cell migration following overexpression of S100A1 in Ishikawa cells. Left: a scratch was made in the middle of a layer of confluent Ish-S100A1 cells or control cells, and phase contrast images were taken 24 h later. The red lines indicate the borderlines between confluent cell layers and wound area. Right: the wound areas were calculated using Image J software version 1.41, with the area at 0 h post-wounding set as 1. The experiments were performed in triplicate. Data are expressed as mean ± SDs. Con, control

**Additional file 9: Table S1.** Relationship between mRNA, copy number variations, and mutations in S100 family members.

**Additional file 10: Table S2.** Relationship between S100A1 score and several clinicopathological factors in endometrial carcinomas.

## Data Availability

All data generated or analyzed during this study are included in this published article and its Additional files.

## Abbreviations

Em Ca: Endometrial carcinoma
MDM2: Mouse double minute 2
KD: Knockdown
cPARP1: cleaved poly (ADP-ribose) polymerase 1
ADR: Adriamycin
HPF: High power filed
IHC: Immunohistochemistry
OS: Overall survival
PFS: Progression free survival
IL: Interleukin

## References

[1] Ryan AJ, Susil B, Jobling TW, Oehler MK. Endometrial cancer. Cell Tissue Res. 2005;322:53–61. 10.1007/s00441-005-1109-5 15947972

[2] Kasoha M, Dernektsi C, Seibold A, Bohle RM, Takacs Z, Ioan-lulian I, et al. Crosstalk of estrogen receptors and Wnt/β-catenin signaling in endometrial cancer. J Cancer Res Clin Oncol. 2020;146:315–27. 10.1007/s00432-019-03114-8 PMC11804341 31865530

[3] Ushijima K (2009). Current status of gynecologic cancer in Japan. J Gynecol Oncol.

[4] Yamagami W, Mikami M, Nagase S, Tabata T, Kobayashi Y, Kaneuchi M (2020). Japan society of gynecologic oncology 2018 guidelines for treatment of uterine body neoplasms. J Gynecol Oncol.

[5] Siegel RL, Miller KD, Jemal A (2016). Cancer statistics. CA Cancer J Clin.

[6] Wang T, Huo X, Chong Z, Khan H, Liu R, Wang T (2018). A review of S100 protein family in lung cancer. Clinica Chimica Acta.

[7] Gonzalez LL, Garrie K, Turner MD (2020). Role of S100 proetins in health and disease. Biochim Biophys Acta Mol Cell Res.

[8] Chen H, Xu C, Jin Q, Liu Z (2014). S100 protein family in human cancer. Am J Cancer Res.

[9] Salama I, Malone PS, Mihaimeed F, Jones JL (2008). A review of the S100 proteins in cancer. Eur J Surg Oncol.

[10] Bresnick AR, Weber DJ, Zimmer DB (2015). S100 proteins in cancer. Nat Rev Cancer.

[11] Ji Y-F, Huang H, Jiang F, Ni R-Z, Xiao M-B (2014). S100 family signaling network and related proteins in pancreatic cancer (Review). Int J Mol Med.

[12] Xu H-Y, Song H-M, Zhou Q (2020). Comprehensive analysis of the expression and prognosis for S100 in human ovarian cancer: A STROBE study. Medicine.

[13] Tochimoto M, Oguri Y, Hashimura M, Konno R, Matsumoto T, Yokoi A (2020). S100A4/non-muscle myosin II signaling regulates epithelial-mesenchymal transition and stemness in uterine carcinosarcoma. Lab Invest.

[14] Hiruta A, Oguri Y, Yokoi A, Matsumoto T, Oda Y, Tomohiro M (2020). S100A4/nonmuscle myosin IIA/p53 axis contributes to aggressive features in ovarian high-grade serous carcinoma. Am J Pathol.

[15] Seagusa M, Hashimura M, Kuwata T (2012). Sox4 functions as a positive regulation of β-catenin signaling through upregulation of *TCF4* during morular differentiation of endometrial carcinomas. Lab Ivest.

[16] Akiya M, Yamazaki M, Matsumoto T, Kawashima Y, Oguri Y, Kajita S (2017). Identification of LEFTY as a molecular marker for ovarian clear cell carcinoma. Oncotarget.

[17] Kerr JF, Winterford CM, Harmon BV (1994). Apoptosis: its significance in cancer and cancer therapy. Cancer.

[18] Zaino R, Carinelli SG, Ellenson LH, Eng C, Katabuchi H, Konishi I, et al. Tumours of the uterine corpus. In: Kurman RJ, Carcangiu ML, Herrington CS, Young RH, editors. WHO classification of tumours of female reproductive organs. Lyon: IARC; 2014. p. 121–54.

[19] Creasman W (2009). Revised FIGO staging for carcinoma of the endometrium. Int J Gynaecol Obstet.

[20] Orre LM, Panizza E, Kaminsky VO, Vernet E, Graslund T, Zhivotovsky B (2013). S100A4 interacts withb p53 in the nucleus and promotes p53 degradation. Oncogene.

[21] Leclerc E, Heizmann CW (2011). The importance of Ca^2+^/Zn^2+^ signaling S100 proteins and RAGE in translational medicine. Front Biosci (Schol. Ed.).

[22] Hermann A, Donato R, Weiger TM, Chazin WJ (2012). S100 calcium binding proteins and ion channels. Front Phamacol.

[23] Donato R, Cannon BR, Sorci G, Riuzzi F, Hsu K, Weber DJ (2013). Functions of S100 proteins. Curr Mol Med.

[24] Matsumoto T, Yoki A, Konno R, Oguri Y, Hashimura M, Tochimoto M (2021). Cytoplasmic EBP50 and elevated PARP1 are unfavorable prognostic factors in ovarian clear cell carcinoma. Carcinogenesis.

[25] Wang G, Rudland PS, White MR, Barraclough R (2000). Interaction *in vivo* and *in vitro* of the metastasis-inducing S100 protein, S100A4(p9Ka) with S100A1. J Biol Chem.

[26] Wang G, Zhang S, Fernig DG, Martin-Fernandez M, Rudland PS, Barraclough R (2005). Mutually antagonistic actions of S100A4 and S100A1 on normal and metastatic phenotypes. Oncogene.

[27] Garbuglia M, Verzini M, Sorci G, Bianchi R, Giambanco I, Agneletii AL (1999). The calcium-modulated proteins, S100A1 and S100B, as potential regulators of the dynamics of type III intermediate filaments. Braz J Med Biol Res.

[28] Terzian T, Suh Y-A, Iwakuma T, Post SM, Neumann M, Lang GA (2008). The inherent instability of mutant p53 is alleviated by *Mdm2* or *p16*^*INK4a*^ loss. Genes Dev.

[29] He M, Rennie PS, Dragiwska V, Nelson CC, Jia W (2002). A mutant p53 can activate apoptosis through a mechanism distinct from those induced by wild type p53. FEBS Lett.

[30] Timofeev O, Klimovich B, Schneikert J, Wanzel M, Pavlakis E, Noll J (2019). Residual apoptotic activity of a tumorigenic p53 mutant improves cancer therapy responses. EMBO J.

[31] van Dieck J, Lum JK, Teufel DP, Fersht AR (2010). S100 proteins interact with the N-terminal domain of MDM2. FEBS lett.

[32] Fernandez-Fernandez MR, Rutherford TJ, Fersht AR (2008). Members of the S100 family bind p53 in two distinct ways. Protein Sci.

[33] van Dieck J, Teufel DP, Jaulent AM, Fernandez-Fernandez MR, Rutherford TJ, Wyslouch-Cieszynska A (2009). Posttranslational modifications affect the interaction of S100 proteins with tumor suppressor p53. J Mol Biol.

[34] Sali A, Glaeser R, Earnest T, Baumeister W (2003). From words to literature in structural proteomics. Nature.

[35] Berge G, Maelandsmo GM (2011). Evaluation of potential interactions between the metastasis-associated protein S100A4 and the tumor suppressor protein p53. Amino Acids.

[36] Ebge M, Bao WJ, Hedstrom E, Jackson SP, Moumen A, Selivanova G (2009). MDM2-dependent downregulation of p21 and hnRNPK provides a switch between apoptosis and growth arrest induced by pharmacologically activated p53. Cancer Cell.

[37] Jin Y, Lee H, Zeng S, Dai MS, Lu H (2003). MDM2 promotes p21waf1/cip1 proteasomal turnover independently of ubiquitylation. EMBO J..

[38] Klein AM, Biderman L, Tong D, Alaghebandan B, Plumber SA, Mueller HS (2021). MDM2, MDMX, and p73 regulate cell-cycle progression in the absence of wild-type p53. Proc Natl Acad Sci USA.

[39] Giese A, Loo MA, Tran N, Haskett D, Coons SW, Berens ME (1996). Dichotomy of astrocytoma migration and proliferation. Int J Cancer.

[40] Giese A, Bjerkvig R, Berens ME, Westphal M (2003). Cost of migration: invasion of malignant glioma and implications for treatment. J Clin Oncol.

[41] Merzak A, McCrea S, Koocheckpour S, Pilkington GJ (1994). Control of human glioma cell growth, migration and invasion *in vitro* by transforming growth factor β1. Br J Cancer.

[42] Tian T, Li X, Hua Z, Ma J, Liu Z, Chen H (2017). S100A1 promotes cell proliferation and migration and is associated with lymph node metastasis in ovarian cancer. Discov Med.

[43] Lu X, Yan C, Huang Y, Shi D, Fu Z, Qiu J (2016). Mouse double minute 2 (MDM2) upregulates Snail expression and induces epithelial-mesenchymal transition in breast cancer cells *in vitro* and *in vivo*. Oncotarget.

[44] Tang Y, Xuan Y, Qiao G, Ou Z, He Z, Zhu Q (2019). MDM2 promotes epithelial-mesenchymal transition through activation of Smad2/3 signaling pathway in lung adenocarcinoma. Onco Targets Ther.

[45] Bai Y, Li L-D, Li J, Lu X (2018). Prognostic values of S100 family members in ovarian cancer patients. BMC Cancer.

[46] DeRycke MS, Andersen JD, Harrington KM, Pambuccian SE, Kalloger SE, Boylan KLM (2009). S100A1 expression in ovarian and endometrial endometrioid carcinomas is a prognostic indicator of relapse-free survival. Am J Clin Pathol.

[47] Kim JH, Oh SH, Kim EJ, Park SJ, Hong SP, Cheon JH (2012). The role of myofibroblasts in upregulation of S100A8 and S100A9 and the differentiation of myeloid cells in the colorectal cancer microenvironment. Biochem Biophys Res Commun.

