# Supplementary figures and images for: Functional interaction between S100A1 and MDM2 may modulate p53 signaling in normal and malignant endometrial cells
Source: BMC Cancer. 2022 Feb 18;22:184. doi: 10.1186/s12885-022-09249-1 (PMC8855586; doi:10.1186/s12885-022-09249-1)

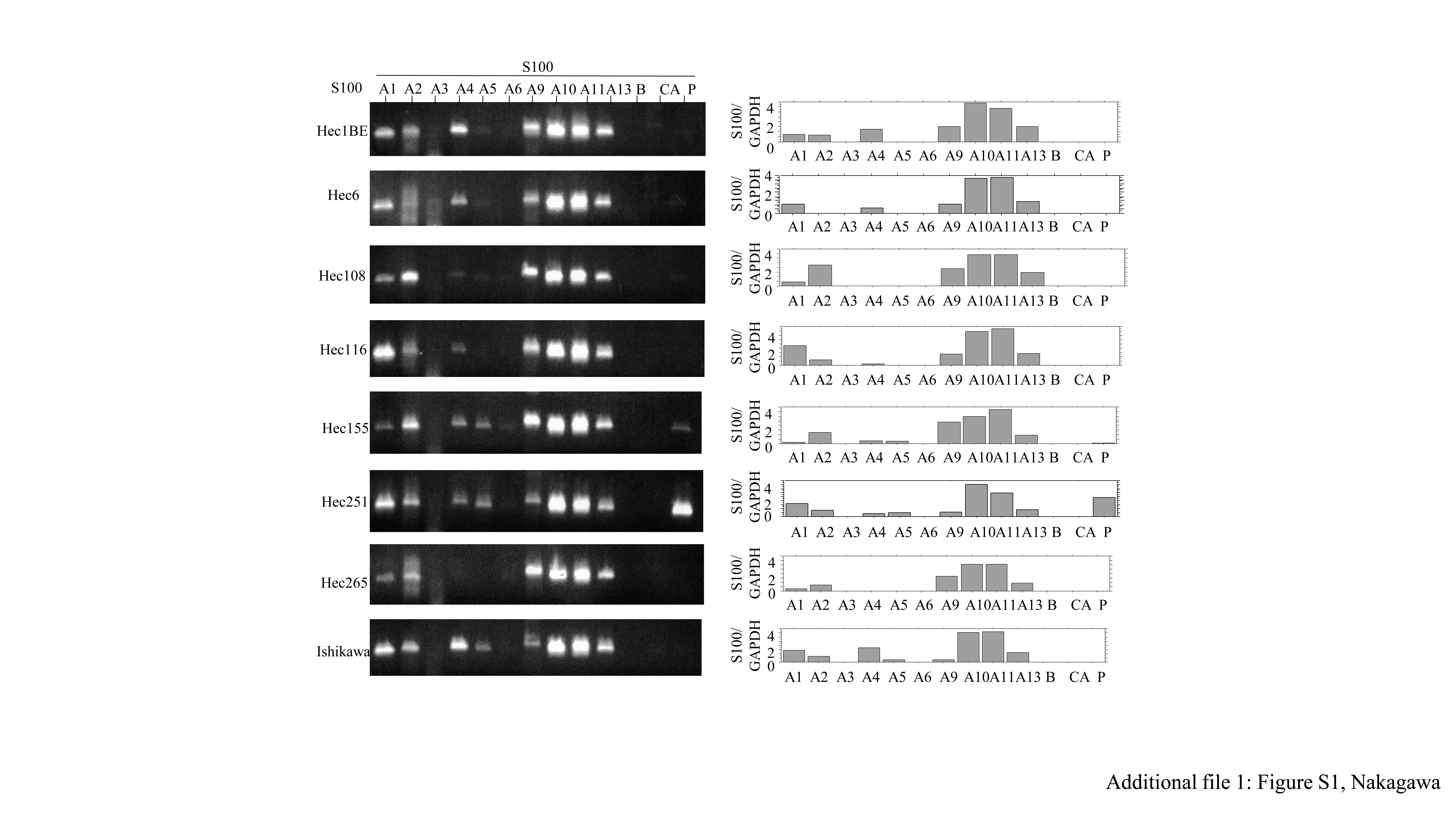

Supplement: Supplementary file 1 — Additional file 1: Figure S1. S100 family member mRNA expression in Em Ca cell lines. Left: expression of 13 S100 family members in eight Em Ca cell lines detected by RT-PCR assay. Right: relative amounts of S100 mRNA calculated by normalization to signals for the GAPDH gene using ImageJ software version 1.41. [file 12885_2022_9249_MOESM1_ESM.tif]

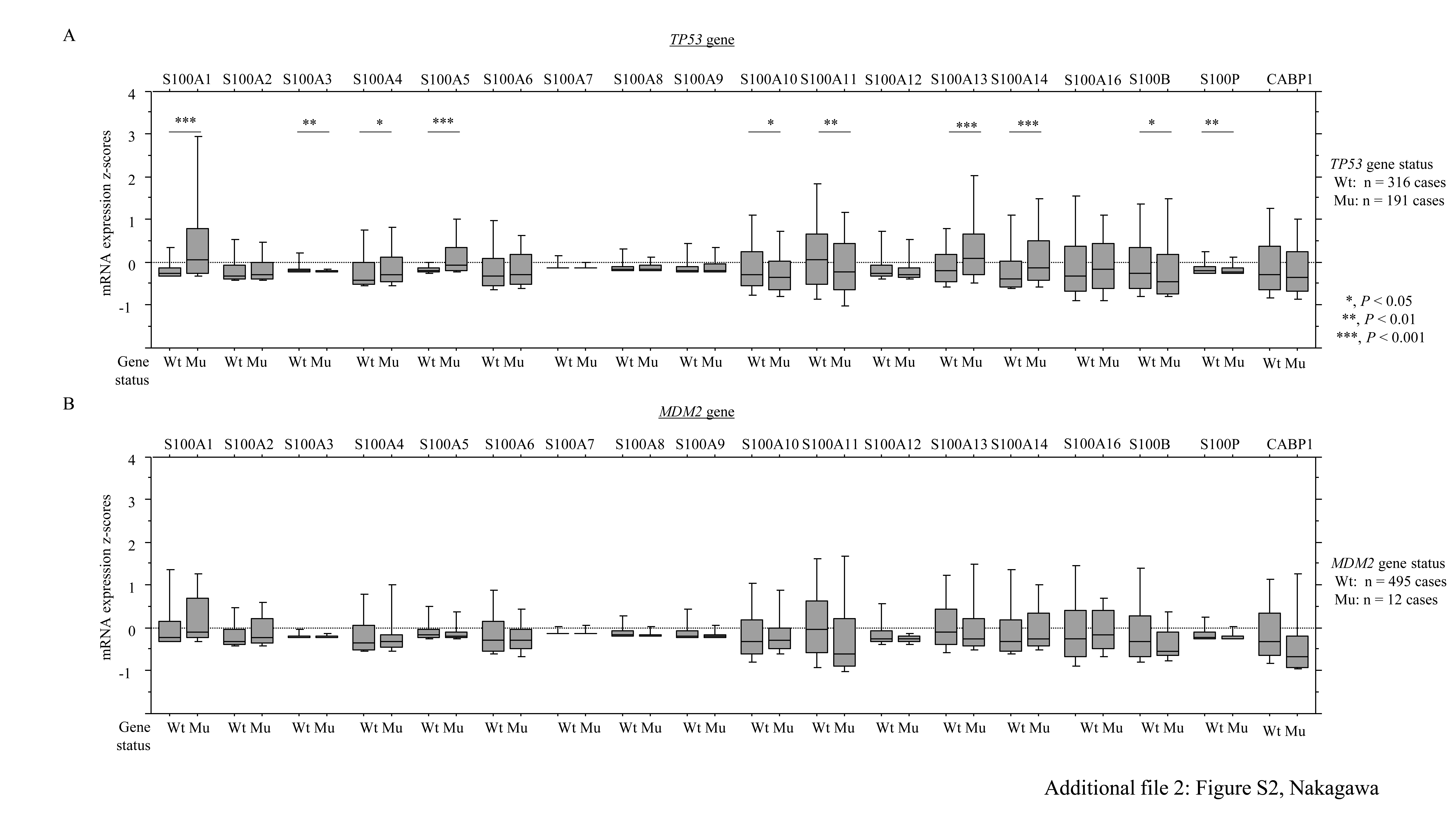

Supplement: Supplementary file 2 — Additional file 2: Figure S2. Relationship between expression of S100 family members and TP53 and MDM2 gene status derived from TCGA Em Ca data analysis. Comparison of the mRNA status of S100 family members between wild- and mutant types of TP53 (A) and MDM2 genes (B). n, number of cases; Wt, wild-type; Mu, mutant type. [file 12885_2022_9249_MOESM2_ESM.tif]

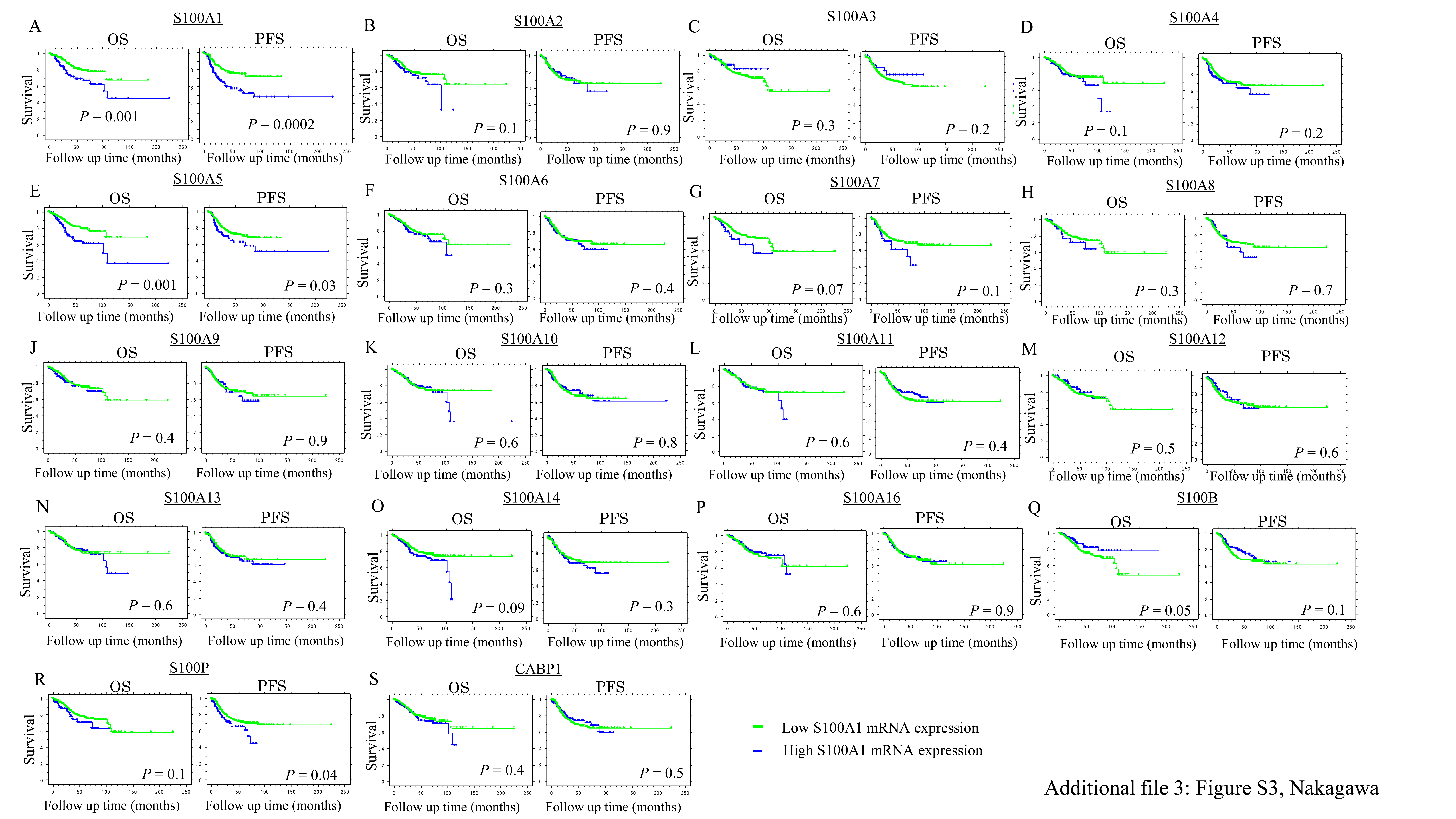

Supplement: Supplementary file 3 — Additional file 3: Figure S3. Relationship between expression of S100 family members and prognosis derived from TCGA Em Ca data analysis. Overall survival (OS) and progression free survival (PFS) between low and high mRNA expression categories of 18 S100 family members. [file 12885_2022_9249_MOESM3_ESM.tif]

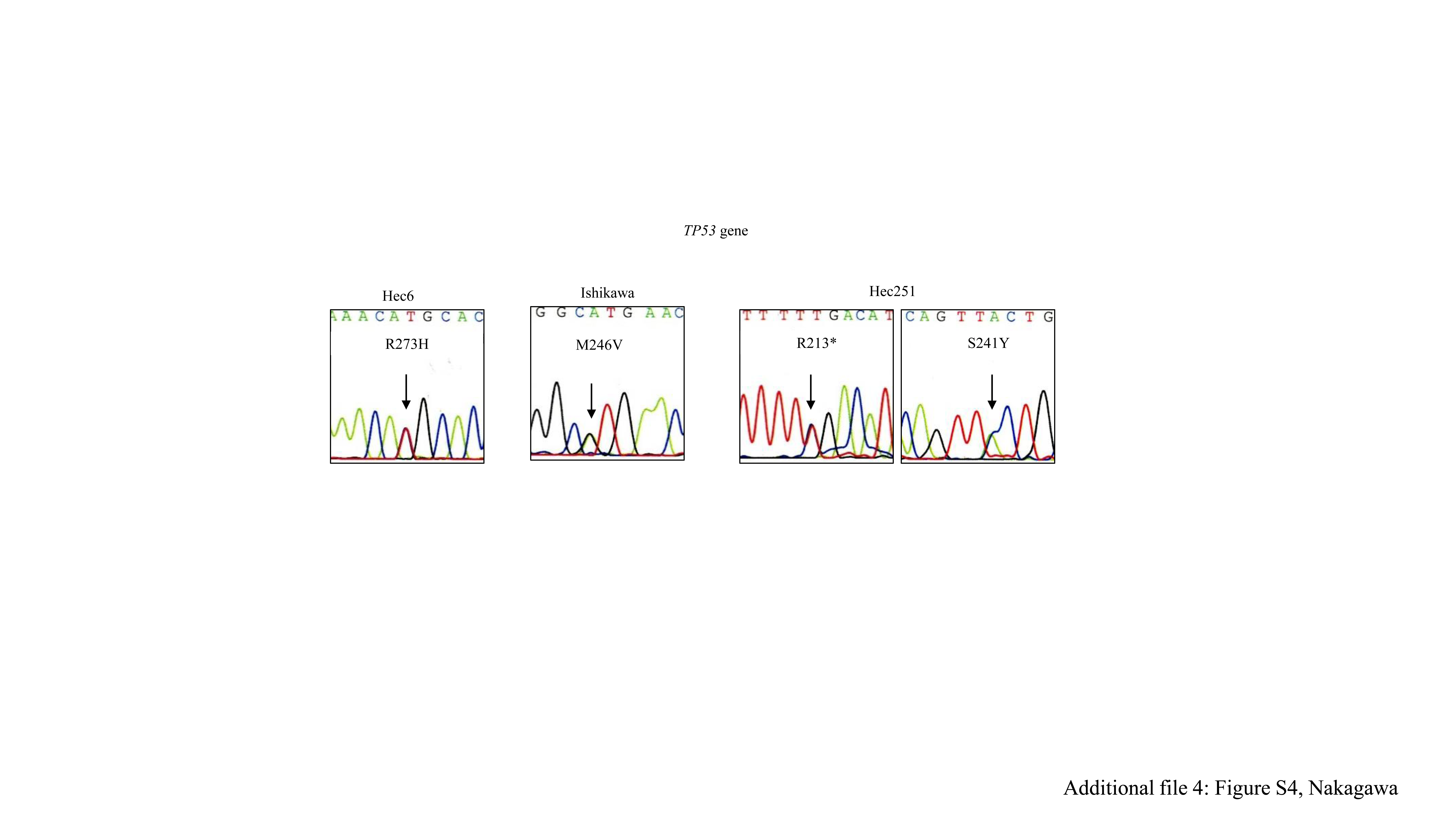

Supplement: Supplementary file 4 — Additional file 4: Figure S4. TP53 gene status in Hec6, Ishikawa, and Hec251 cell lines. *, stop codon. [file 12885_2022_9249_MOESM4_ESM.tif]

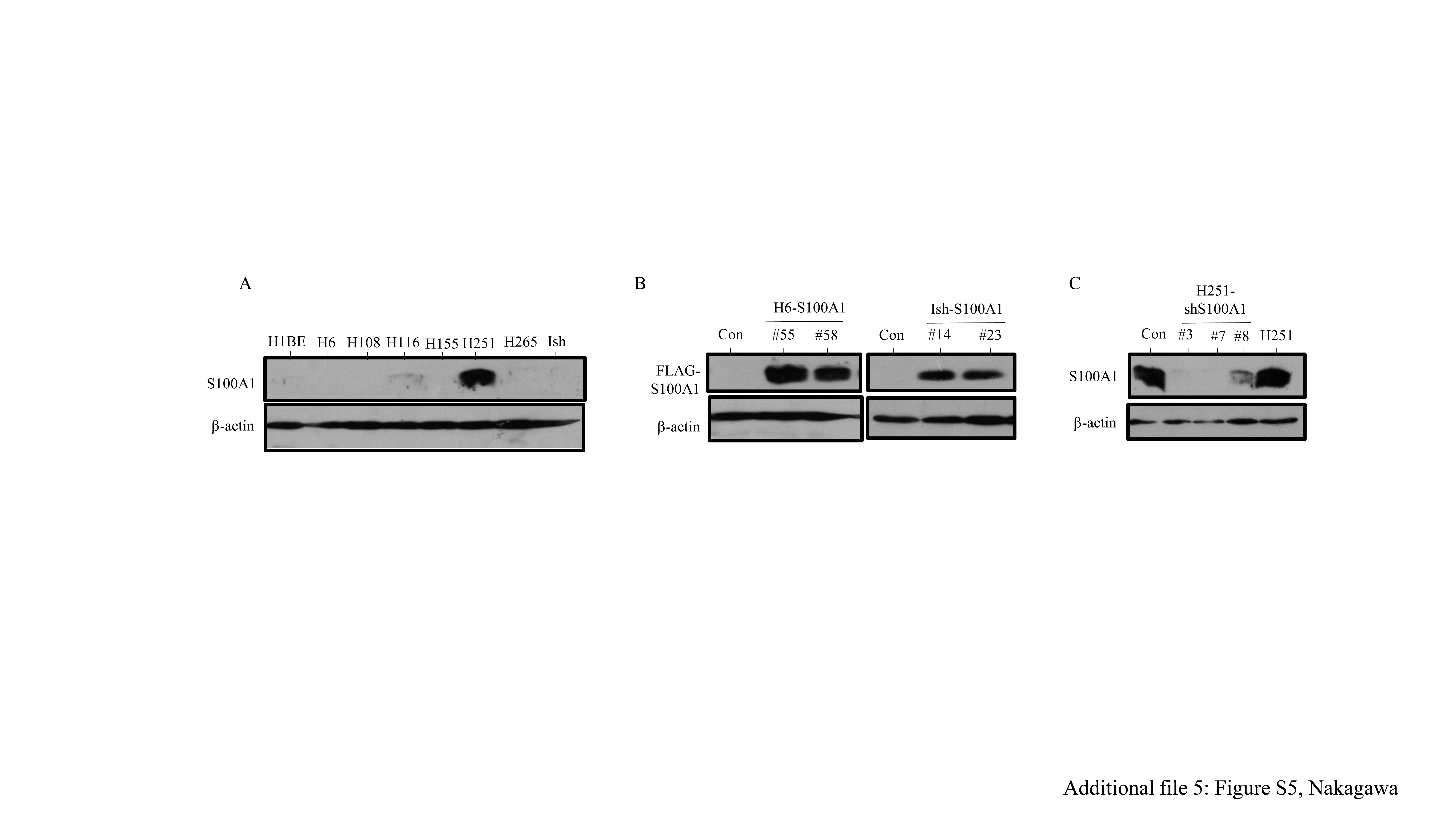

Supplement: Supplementary file 5 — Additional file 5: Figure S5. Western blot analysis of the indicated proteins in total lysates from eight Em Ca cell lines (A), stable H6- and Ish-S100A1 cells (B), and H251-shS100A1 cells (C), as well as control cells (Con). [file 12885_2022_9249_MOESM5_ESM.tif]

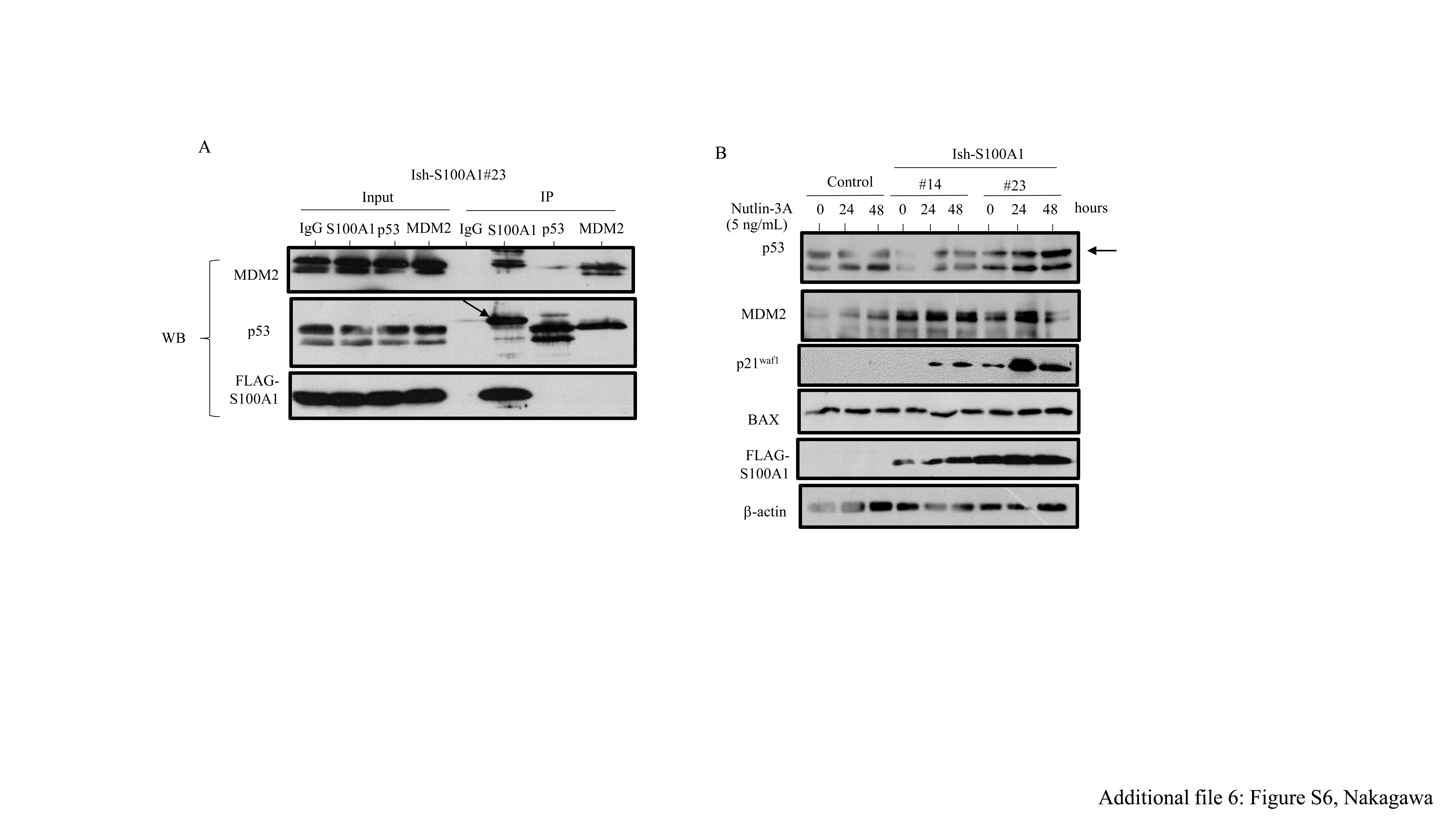

Supplement: Supplementary file 6 — Additional file 6: Figure S6. Interactions between S100A1, MDM2, and p53 in stable Ish-S100A1 cells. (A) Western blot analysis (WB) with anti-MDM2 (upper panel), anti-p53 (middle panel), and anti-FLAG M2 antibodies (lower panel) after immunoprecipitation (IP) with the indicated antibodies using stable Ish-S100A1 cell lysates. Input represents 5% of the total cell extract. Normal rabbit IgG was used as a negative control. In the middle panel (p53), the band indicated by an arrow in the S100A1 lane is non-specific, since the molecular weight is slightly higher compared to that of endogenous p53. (B) Western blot analysis of the indicated proteins in stable Ish-S100A1 cells in response to Nutlin-3A treatment for the times shown. The p53 band is indicated by arrows. [file 12885_2022_9249_MOESM6_ESM.tif]

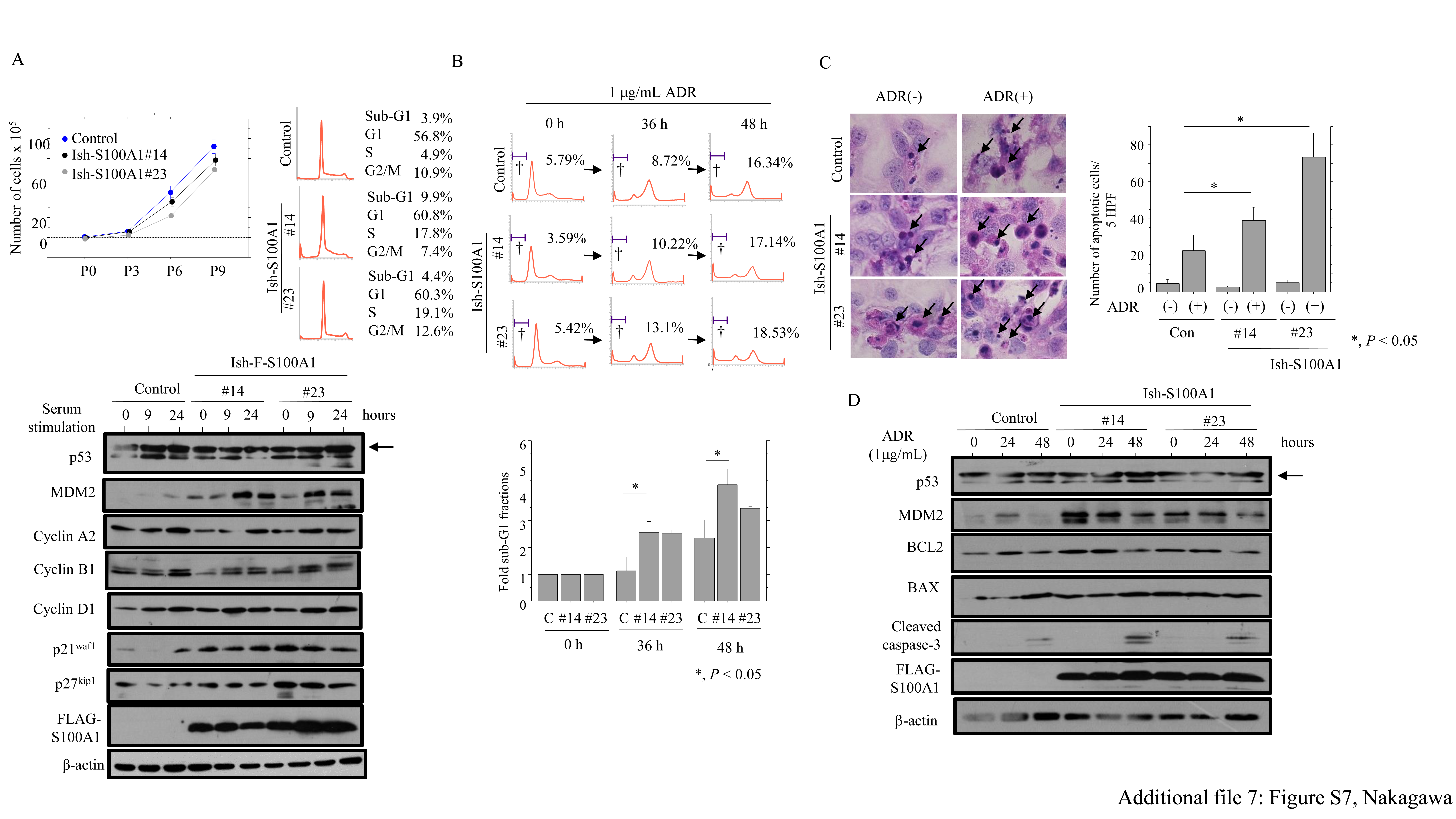

Supplement: Supplementary file 7 — Additional file 7: Figure S7. Changes in proliferation and apoptosis following overexpression of S100A1 in Ishikawa cells. (A) Upper left: two independent clones of stable Ish-S100A1 cells and control cells were seeded at low density. The cell numbers are presented as mean ± SDs. P0, P3, P6, and P9 are 0, 3, 6, and 9 days after cell passage, respectively. Upper right: FACS analyses of stable Ish-S100A1 and control cells at 3 days after seeding (P3). Lower: western blot analysis of the indicated proteins in stable Ish-S100A1 cells and controls following re-stimulation of serum-starved (24 h) cells with 10% serum for the indicated times. The p53 band is indicated by arrows. (B) Upper: stable Ish-S100A1 and control cells were treated with 1 μg/mL Adriamycin (ADR) for the times shown. Daggers indicate the sub-G1 fraction. Lower: the percentages of cells undergoing apoptosis (sub-G1 fractions) were calculated following flow cytometry. C, control (C) Left: after 1 μg/mL ADR treatment, stable Ish-S100A1 and control cells undergoing apoptosis are indicated by arrows. Original magnification, x400. Right: the numbers of apoptotic cells are shown as mean ± SDs. Con, control (D) Western blot analysis of the indicated proteins in total lysates from stable Ish-S100A1 and control cells treated with 1 μg/mL ADR for the times shown. The p53 band is indicated by arrows. [file 12885_2022_9249_MOESM7_ESM.tif]

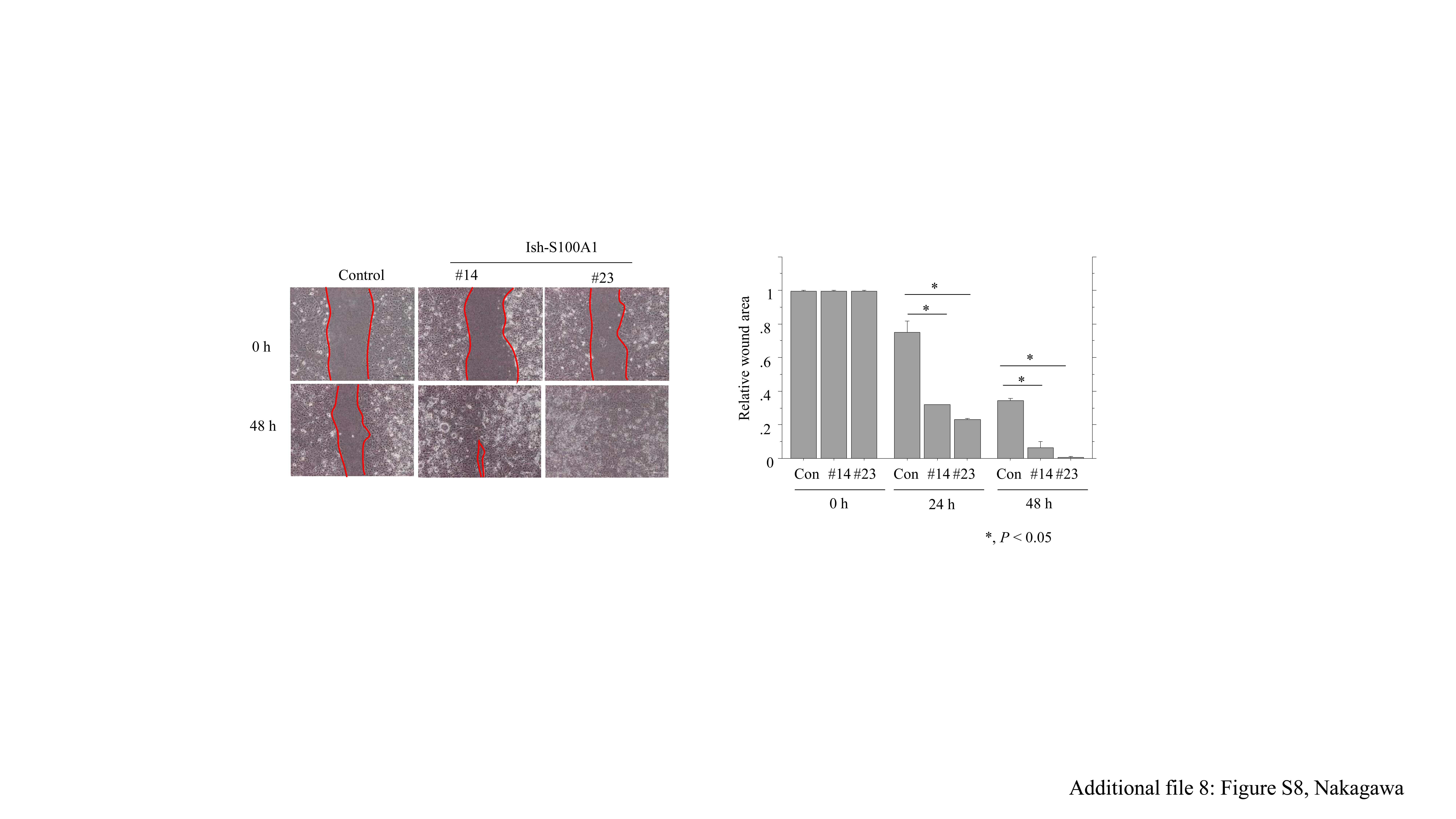

Supplement: Supplementary file 8 — Additional file 8: Figure S8. Changes in cell migration following overexpression of S100A1 in Ishikawa cells. Left: a scratch was made in the middle of a layer of confluent Ish-S100A1 cells or control cells, and phase contrast images were taken 24 h later. The red lines indicate the borderlines between confluent cell layers and wound area. Right: the wound areas were calculated using Image J software version 1.41, with the area at 0 h post-wounding set as 1. The experiments were performed in triplicate. Data are expressed as mean ± SDs. Con, control [file 12885_2022_9249_MOESM8_ESM.tif]
